# A Custom-Made Orthodontic Mini-Implant—Effect of Insertion Angle and Cortical Bone Thickness on Stress Distribution with a Complex In Vitro and In Vivo Biosafety Profile

**DOI:** 10.3390/ma13214789

**Published:** 2020-10-27

**Authors:** Adelina Popa, Cristina Dehelean, Horia Calniceanu, Claudia Watz, Silviu Brad, Cosmin Sinescu, Olivia A. Marcu, Casiana Simina Popa, Stefana Avram, Mirela Nicolov, Camelia A. Szuhanek

**Affiliations:** 1 2nd Department of Orthodontics, Faculty of Dental Medicine, Victor Babes University of Medicine and Pharmacy, 300041 Timisoara, Romania; popa.adelina@umft.ro (A.P.); cameliaszuhanek@umft.ro (C.A.S.); 2 2nd Department of Toxicology and Drug Industry, Faculty of Pharmacy, Victor Babes University of Medicine and Pharmacy, 300041 Timisoara, Romania; cadehelean@umft.ro; 3 1st Department/Periodontology, Faculty of Dental Medicine, Victor Babes University of Medicine and Pharmacy, 300041 Timisoara, Romania; 4 1st Department of Pharmaceutical Physics and Biophysics, Faculty of Pharmacy, Victor Babes University of Medicine and Pharmacy, 300041 Timisoara, Romania; nicolovmirela@gmail.com; 5 2nd Department of Radiology, Faculty of Dental Medicine, Victor Babes University of Medicine and Pharmacy, 300041 Timisoara, Romania; bradsilviu@yahoo.com; 6 2nd Department of Prostheses Technology and Dental Material, Faculty of Dental Medicine, Victor Babes University of Medicine and Pharmacy, 300041 Timisoara, Romania; minosinescu@yahoo.com; 7 Dental Medicine Department, Faculty of Medicine and Pharmacy, University of Oradea, 410087 Oradea, Romania; oli_baciu@yahoo.com; 8 PAN Dental X RAY, 310337 Arad, Romania; Cassiana_popa@yahoo.com; 9 2nd Department of Pharmacognosy, Faculty of Pharmacy, Victor Babes University of Medicine and Pharmacy, 300041 Timisoara, Romania; stefana.avram@umft.ro

**Keywords:** orthodontic mini-implants, insertion angle, cortical bone thickness, finite element analysis, primary stability, HGF, in vitro cytotoxicity, HET-CAM assay

## Abstract

Background: Orthodontic mini-implant failure is a debatable subject in clinical practice. However, the most important parameter to evaluate the success rate of mini-implant is the primary stability, which is mainly influenced by cortical bone thickness (CBT) and insertion angle. Materials and methods: Three-dimensional finite element models of the maxilla were created and a custom-made, self-drilling, tapered mini-implant was designed. For the pull-out test, 12 simulations were performed, sequentially increasing the thickness of the cortical bone (1, 1.5 and 2 mm) and the insertion angle (30°, 60°, 90°, 120°). For the force analysis, 24 simulations were performed using an experimental orthodontic traction force of 2 N both in the horizontal and vertical axis. Results: Insertion angle and CBT have significant impact on force reaction values (*p* < 0.05). Cortical bone stress had the lowest value when the mini-implant had a 30° insertion angle and the highest value when the implant had a 120° insertion angle, while the CBT was 1 mm. Cortical bone stress had the lowest value with an insertion angle of 90° and the highest value when the implant was inserted at an angle of 30°, while the CBT was 2 mm independent of the force direction. Regarding the biosafety profile of the mini-implant alloy, the present results reveal that the custom-made mini-implant presents good biocompatibility. Conclusions: When the CBT is reduced, we recommend inclined insertion while, when the CBT is appropriate, perpendicular insertion is advised.

## 1. Introduction

Anchorage is one of the major challenges for orthodontists. Orthodontic mini-implants are now widely used, even if there is no universally accepted design and insertion protocol. The reported failure rate is modest (13.5%), which suggests that mini-implants are clinically reliable orthodontic devices [1]. However, several complications have been reported in the literature, such as damage to the anatomical structures, inflammation or even peri-implantitis and subsequent failure of the orthodontic treatment [2]. Three main group factors may affect the mini-implant success rate, which are as follows: patient-dependent aspects, mini-implant features or technique-dependent factors [3].

The primary stability of mini-implants is considered the most important criterion to assess the success rate of orthodontic mini-implants. It is defined as the result of the mechanical interlocking between the mini-implant with the surrounding bone, which is determined by several factors: cortical bone quality and quantity, operator technique and the diameter of the screw [4,5]. However, primary stability is also influenced by implant design and surface treatment [6]. Nevertheless, from a mechanical point of view, in order to achieve good primary stability, the goal of mini-implant placement is to obtain maximum inter-digitation between the bone tissue and the threads while generating controlled compression forces in the bone [7]. Placement sites with greater cortical bone thickness (CBT) and higher cortical bone density are ideal and have been shown to contribute to the success rates [8]. From a biomechanical perspective, CBT values in the range of 1.0 to 2.0 mm appear to be appropriate for orthodontic mini-implant therapy [9]. The optimal insertion angle of a mini-implant is important for cortical anchorage and there are different opinions regarding this aspect. Several studies reported that placement of the mini-implant perpendicular to the long axis of the tooth offers more stability to orthodontic loading [10,11]; others suggest that an oblique insertion reduces stress on the mini-implant [12] and engages more of the cortical bone, thus increasing primary stability, while Lin et al. suggested that orthodontic force direction has no significant effect on cortical bone stress [13].

To achieve an analytical solution for problems involving complicated geometries, such as the maxilla and mandible, which are exposed to various kinds of loads is quite difficult [10]. Moreover, it is virtually impossible to measure mini-implant and bone stress accurately in vivo. To overcome the above-mentioned limitations, the finite element method (FEM)—a modern analytical technique of measuring numerical stress—was developed. This innovative method has the advantage of being applicable to solids of irregular geometry that contain heterogeneous material properties and it also provides an approximate solution for the response of three-dimensional (3D) structures to external loads applied under certain boundary conditions [14].

Besides the primary stability, the success rate of an orthodontic mini-implant is also related to the chemical composition of the metal alloy from which it is made and which must possess some essential features—in particular, biocompatibility/non-cytotoxicity—to be further approved for clinical use. Several studies support the release of metal ions from orthodontic implants [15,16]; thereby, this aspect becomes even more important in the case of a custom-made mini-implant. Concerning this issue, it is of significant importance to assess the biocompatibility of the metal alloy proposed for the development of the custom-made mini-implant on buccal cells through basic cytotoxic methods, such as 3-(4,5-dimethylthiazol-2-yl)-2,5-diphenyltetrazolium bromide (MTT) assay and lactate dehydrogenase (LDH) release method, which are the most used in vitro colorimetric assays to evaluate the toxicity/cytocompatibility of a test sample. Moreover, to complete the safety profile of the designed mini-implant next to the in vitro models, in vivo protocols often can add features linked to the complexity of a biological system. For further prediction of the biocompatibility designed mini-implant, an in vivo evaluation can add relevant data to its safety profile. The hen’s egg test on chorioallantoic membrane (HET-CAM) assay is a simple and versatile alternative to animal testing [17]. This method also provides a rapidly growing vascular bed, devoid of a nervous system, with a slow immune system [18] and hence an alternative as a preliminary in vivo pre-clinical evaluation tool. Similar studies have employed the same methods or variations of these to assess the biosafety profile of orthodontic devices [15,19,20].

The aim of the present study is to evaluate the stability of a custom-made mini-implant using 3D finite element models and to validate its biosafety profile using primary human gingival fibroblasts (HGF cells) for further in vivo clinical application.

## 2. Materials and Methods

**Figure 1:**
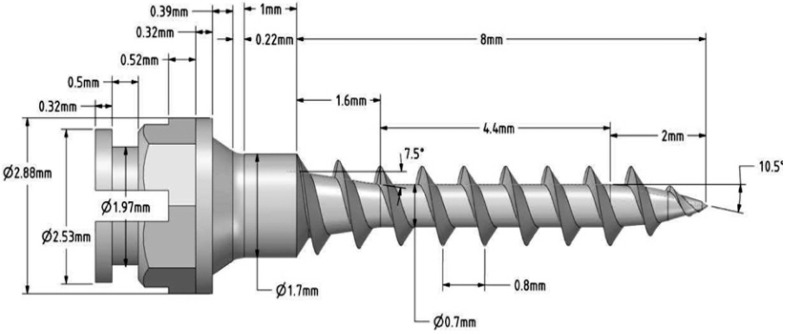
Three-dimensional rendering of the custom-made mini-implant design.

Three-dimensional finite element models of the maxilla were created after cone beam computed tomography scanning with a slice of 1.5 mm (Kavo OP 3D Pro, FOV 6 × 8 cm, scan time: 1.7 s, radiation dose: 8 µSv, OnDemand3D™ software). Following the scanning, the morphological data were manually segmented using the Materialise Mimics InPrint software 3.0. Segmented models of the cortical and cancellous bone and the crowns of teeth 24, 25, 26 and 27 were imported into ANSYS Space Claim 2017.1. A custom-made, self-drilling, tapered mini-implant was designed using FreeCad 0.18 software. The implant had a diameter of 1.7 mm, 8 mm in length, 0.8 mm pitch, 0.32 mm thread depth and the bracket-like head from Forestadent (Ortho Easy, Pforzheim, Germany), as presented in Figure 1.

The implant was inserted into the most popular insertion site: the inter-dental space between the upper first molar and second premolar [21,22,23].

**Figure 2:**
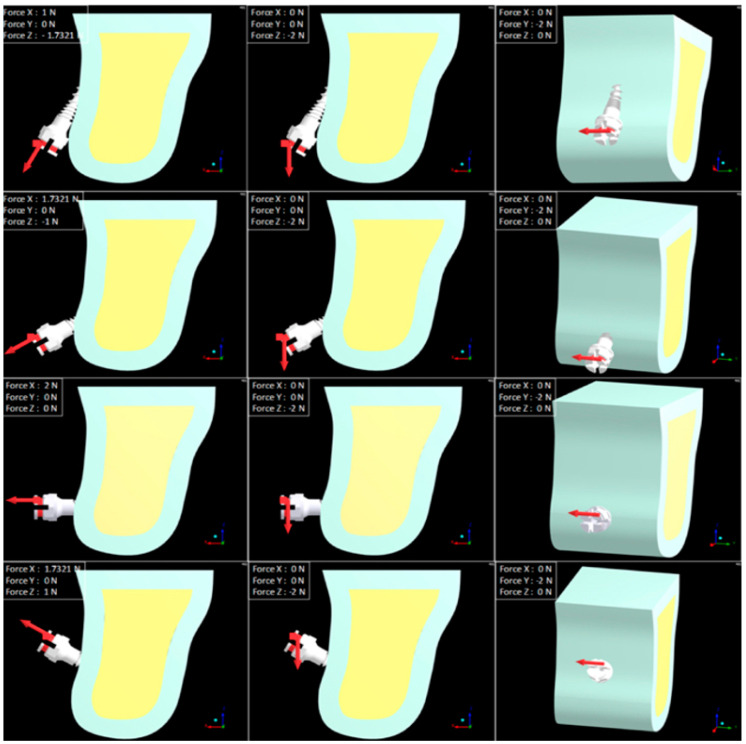
Insertion angles and force directions.

Cortical bone was modeled with a 1 mm thickness for the first set of simulations; afterwards, the thickness increased to 1.5 and 2 mm for the following simulations. CBT values were selected based on published data on human jawbone structure [24,25]. Mini-implant insertion angles were 30°, 60°, 90°, 120° [10,11,13,26,27] and the pull-out test was simulated until the axial displacement of the implant was 0.01 mm [28]. An experimental orthodontic traction force of 2 N was applied to the head of each implant both in the horizontal and in the vertical direction in order to simulate en-masse retraction and intrusion (Figure 2). An orthodontic force of 2 N (200 g) is in the generally accepted range according to the reported clinically safe limit for immediate loading of a mini-implant [12,29,30,31,32].

The materials were considered to be linear, elastic, homogenous (the elastic properties were the same at all points in the material) and isotropic (the same elastic properties existed in all directions at any point in the material) [29,33,34,35]. All the elements were considered bonded [36]. The Young modulus for titanium screw and cortical and cancellous bone were assumed to be 110,000, 13,700 and 1600 MPa, respectively, and Poisson’s ratio was considered 0.35, 0.30 and 0.30, respectively [10]. A mesh of quadratic 10-node tetrahedral structural solid element (C3D10), that is optimized for use in contact analyses, was considered before applying each mini-implant and bone block model. Tetrahedral elements are quadratic and not linear, providing greater precision. They can be used for larger deformations and the total number of elements is reduced due to additional nodes at each half of the element [21]. Before analysis, various element sizes, ranging from 0.01 to 1 mm, were examined to ensure mesh independency of the finite element model. Special care was paid to make sure that the complex geometry of the mini-implant threads and the interfacial bones preserved their original shape without distortion induced by meshing errors. By choosing a finer mesh of 0.1 mm for the mini-implant, each implant consisted of approximately 253.306 nodes and 172.508 solid elements. By choosing a mesh of 0.5 mm for the bone (cortical and cancellous), each cortical bone consisted of 235.573 nodes and 155.736 solid elements and each cancellous bone consisted of 219.322 nodes and 153.980 solid elements. Compared to other similar research, the meshes used for the mini-implant and the bone were finer [9] and the number of nodes and elements was increased [37,38].

The fixed support boundary conditions were applied on the upper side of the assembly and it constrained all 6 DOFs (degrees of freedom). Additional boundary conditions were applied to the screw to restrain movement in all directions except for the direction of the applied force.

For the pull-out test, the second boundary applied the axial displacement of 0.01 mm along the axis of the screw [28]; all the other axes were blocked and rotations were not constrained. The first set of analysis had 12 models created by increasing the CBT (1, 1.5 and 2 mm) and the insertion angle (30°, 60°, 90°, 120°).

For the force analysis, the second boundary condition was the 2 N force that was applied on the Y axis in the first case and the Z axis in the second case. An total of 24 simulations were performed, increasing CBT (1, 1.5 and 2 mm) and the angle of insertion (30°, 60°, 90°, 120°) and changing force orientation (horizontal and vertical). Each analysis was run and the peak von Mises (equivalent) stresses were extracted.

For the pull-out test, force reaction was determined by varying the CBT and insertion angle. The pull-out test is the most commonly used parameter for quantifying the stability of mini-implants, and a higher value suggests increased stability.

The von Mises stress of the mini-implant and the bone surrounding it was determined for the force analysis by varying the CBT, insertion angle and force direction. Mainly, there are three types of stress: tensile, compressive and shear stresses. The von Mises stress is widely used to determine whether the design withstands the given loaded condition [39,40]. The numerical data were calculated and a color band diagram was created for a better understanding of the mechanical phenomena in models. The stress values were measured in mega Pascal (MPa). A lower von Mises stress suggests that the stress on the bone surrounding the implant is lower, the possibility of being damaged is lower and the success rate is higher.

### 2.1. In Vitro Model

The cell line used in the current study consisted of a primary human gingival fibroblast (HGF) monolayer. The cells were supplied by American Type Culture Collection (ATCC^®^ PCS-201-018™, Manassas, VA, USA), together with the culture medium—Fibroblast Basal Medium (ATCC^®^ PCS-201-030™)—and the required supplements—Fibroblast Growth Kit-Low Serum (ATCC^®^ PCS-201-041™) and 0.1% Penicillin-Streptomycin-Amphotericin B Solution (ATCC^®^ PCS-999-002™). The cells were cultured under sterile conditions and humidified atmosphere, enriched with 5% CO_2_ (Steri-Cycle i160 incubator; Thermo Fisher Scientific, Waltham, MA, USA).

The method employed in the present study to assess the in vitro cytocompatibility profile of the Ti-6Al-V metal alloy used for the development of the custom-made orthodontic mini-implant was based on the extraction means, a technique suggested by ISO standard 10993-5:2009 [41] for medical devices. Thus, test mini-implant alloy was submerged into the culture medium and exposed to intermittent shaking for 24 h. Afterwards, the sample was removed and the resulting extraction medium was used to stimulate the HGF cells for different time intervals (24, 48, 72 h).

### 2.2. Cell Morphology Assessment

The possible morphological changes in HGF cells after exposure to the extraction medium were assessed by comparing photographs between control cells (un-stimulated cells) and treated cells. All pictures were taken at magnification 10×, using an Olympus IX73 inverted microscope documented with DP74 camera (Olympus, Tokyo, Japan).

### 2.3. In Vitro Colorimetric Assays—3-(4,5-Dimethylthiazol-2-yl)-2,5-Diphenyltetrazolium Bromide (MTT) Assay and Lactate Dehydrogenase (LDH) Release Method

The protocols employed for the two in vitro colorimetric assays are quite similar; however, the principle of each technique is different. The MTT assay evaluates the metabolically active cells through mitochondrial dehydrogenase activity, while the LDH method assesses the extracellular release of the LDH cytosolic enzyme, which occurs especially if the cellular membrane is damaged, an indicator of cytotoxic activity. 

In brief, the protocol consisted of culturing the cells to a density of 10^4^ cells/well in 96-well plates and incubation overnight. On the following day, the cells were treated with the extraction medium for different intervals of time (24, 48, 72 h). At the end of each stimulation interval, the MTT/LDH protocol provided by the manufacturer was employed.

The optical density (O.D.) of each well was determined spectrophotometrically by means of a microplate reader (xMark™ Microplate; Bio-Rad Laboratories, Hercules, CA, USA).

### 2.4. In Vivo Biocompatibility Assessment/Vascular Toxicity Using the HET-CAM Model

Extracted medium was evaluated using a slightly modified HET-CAM protocol [19,42,43]. The fertilized eggs were incubated at 37 °C and 60% humidity and were prepared according to the standard protocol [44,45]. On day 9 of incubation, inside an opening performed on the eggs’ shell, 200 µL of the extraction medium was inoculated onto the displaying CAM vessels. The effect on the developing capillaries was assessed by monitoring the appearance of hemorrhage (H), lysis (L) or coagulation (C). Further, an irritation score (IS) was calculated using the following equation.

(1) $$\mathrm{IS}\;=\;5\;\times \;\frac{301-\mathrm{Sec}\;H}{300}\;+\;7\;\times \;\frac{301-Sec\;L}{300}\;+\;9\;\times \;\frac{301-Sec\;C}{300}$$

As controls, sodium laurylsulphate (SLS 0.5%) was used as irritative standard, while distilled water was used as the negative control. A follow-up of the tested specimens was also registered 24 and 48 h after the inoculation. Evaluation was performed under stereomicroscopic (Discovery 8 Stereomicroscope, Zeiss, Göttingen, Germany) observation, and relevant photographs of the CAM surface were recorded using the attached camera (Axio CAM 105 color, Zeiss, Göttingen, Germany). All captures were then processed using Zeiss ZEN software, Gimp 2.8 (https://www.gimp.org/) and ImageJ v 1.50e software.

### 2.5. Statistical Analysis

Two analyses were performed, one for the pull-out test and the other one for the force.

A model with two independent factors and force reaction as the dependent variable was developed for the pull-out test results. Insertion angle and CBT were considered the independent factors. A total of 12 models were generated (3 CBT and 4 insertion angles). All the statistical analysis was performed using the SPSS 24 statistical package.

A second model, with three independent factors, was developed to assess the maximum stress of the mini-implant and bone surrounding it using the von Mises variable. A total of 24 models were generated (3CBT, 4 insertion angles and 2 directions of force). The stress of the implant was assessed both at the level of the body and at the level of the head, and the bone was evaluated separately (cortical and cancellous).

## 3. Results

The ANOVA results of the independent factors for the pull-out test are listed in Table 1. Insertion angle and CBT have a significant impact on force reaction values (*p* < 0.05). When CBT increases (from 1 to 1.5, 1 to 2 or 1.5 to 2 mm), force reaction increases as well.

The mean plots and Scheffe’s results demonstrate that there is a significant change in force reaction when the insertion angle is changed. Force reaction values decreased gradually when increasing the insertion angle (30° to 60° to 90°), and the value increased again in conjunction with increases in the insertion angle (90° to 120°).

The results of the ANOVA factorial design of the insertion angle, CBT and force direction and their interactions with the von Mises variable on mini-implant body, mini-implant head, cortical and cancellous bone are presented in Table 2.

Insertion angle, CBT and force direction have a significant impact on the von Mises stress level of the implant and the cancellous bone. Changing the direction of the force from horizontal to vertical leads to a decrease in the von Mises stress level of the implant. There are statistically significant changes in stress levels in both implant and cancellous bone when the CBT is increased. The combined effects of force direction and insertion angle and of insertion angle and CBT are statistically significant in both implant and cancellous bone.

**Figure 3:**
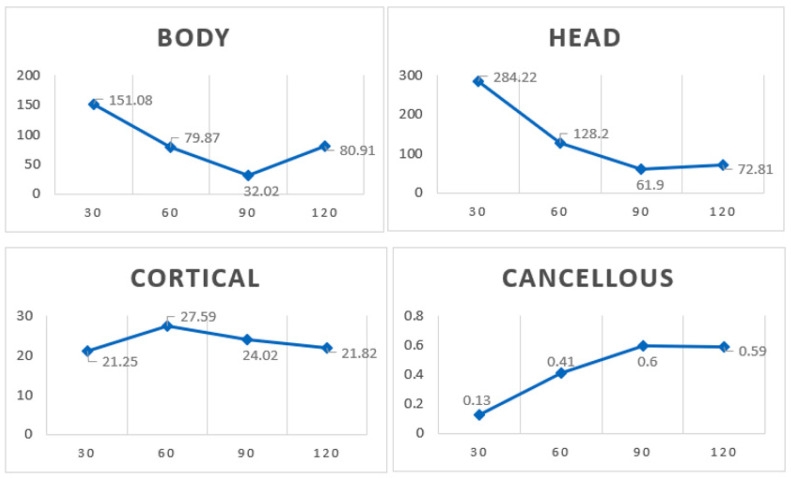
Mean von Mises stress level variation with the different insertion angles.

The variation of the mean von Mises variable with different insertion angles, independent of force direction and CBT, is shown in Figure 3. Based on Scheffe’s results and by inspecting the plots, when the insertion angle is changed from 30° to 60°, 90° or 120° degrees, all changes are statistically significant.

**Figure 4:**
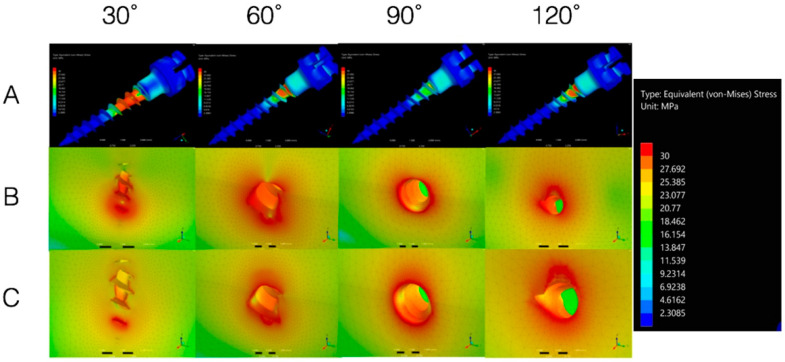
(**A**) Mini-implant von Mises stress; (**B**) Cortical bone von Mises stress with CBT of 1 mm; (**C**) Cortical bone stress with CBT of 2 mm.

Stress decreased at the mini-implant level when the insertion angle increased from 30°, 60° to 90° but it increased again at a 120° insertion angle, when the traction force was horizontal (Figure 4A).

Cortical bone stress had the lowest value with an insertion angle of 30° and the highest value when the implant was inserted at a 120° angle, while CBT was 1 mm (Figure 4B).

Cortical bone stress had the lowest value with an insertion angle of 90°and the highest value when the implant was inserted at a 30° angle, while CBT was 2 mm independent of the force direction (Figure 4C).

### 3.1. In Vitro Biological Profile

We carried out morphological evaluation of primary human gingival fibroblasts (HGF) and evaluation of HGF cells viable/death population.

**Figure 5:**
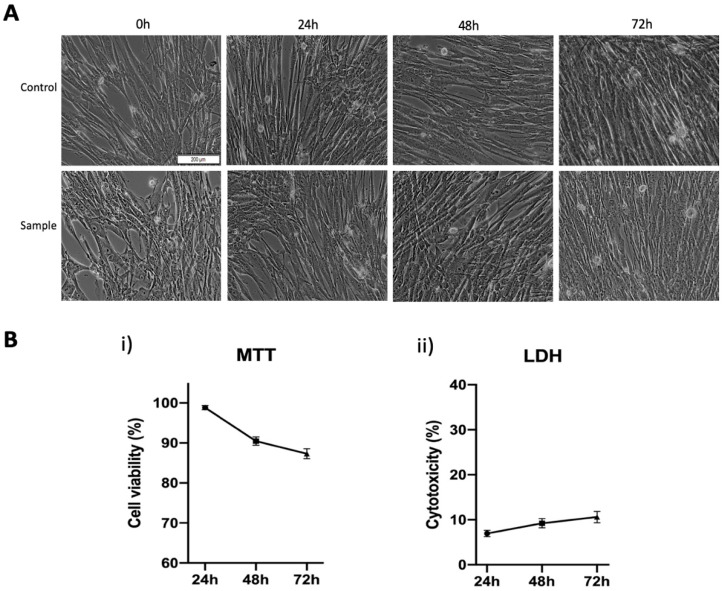
(**A**) Morphological aspects of primary human gingival fibroblasts exposed to the sample (metal alloy of the custom-made mini-implant). The scale bar represents 200 µm; (**B**) (**i**) Cell viability percentage of primary human gingival fibroblasts (HGF) after treatment with the extraction medium (**ii**) Cytotoxic rate of primary human gingival fibroblasts (HGF) at 24, 48 and 72 h post-treatment with extraction medium of test alloy intended for orthodontic mini-implant.

To observe the possible morphological alterations that may be induced by the metal alloy of the custom-made mini-implant, the human gingival fibroblasts were treated with the corresponding extraction medium and were periodically supervised by taking pictures initially (0 h) and at different time intervals (24, 48, 72 h). The results are presented in Figure 5A. Further, the biosafety of the custom-made mini-implant was assessed by employing an in vitro model based on primary human gingival fibroblasts (HGF). In this regard, two different colorimetric methods were performed to evaluate the viability of the stimulated cells and the cytotoxic rate induced by the extraction medium on the HGF population (Figure 5B(i,ii)).

As depicted in Figure 5A, the morphological features (elongated cells with a spindle-like shape) of the primary human gingival fibroblasts treated with the extraction medium of the custom-made mini-implant alloy are very similar to those of the un-stimulated cells (control cells—treated with cell culture medium). Moreover, the initial (0 h) confluence of the treated cells demonstrates the same pattern as the control cells even at consistent time intervals (24, 48, 72 h).

As presented in Figure 5B(i), HGF cells present a good viable population after exposure to the extraction medium of the test sample, the cells manifesting a time-dependent cell viability decrease as follows: cell viability above 98% after 24 h post-stimulation, approximately 90% viable cells after an exposure time of 48h and a viable HGF population around 87% at 72 h post-treatment. The LDH release method showed similar results (Figure 5B(ii)) with the ones obtained in the case of the MTT method. Accordingly, in terms of cytotoxicity, cell death also increased in a time-dependent manner, the cells expressing a cytotoxic rate of approximately 7% after 24 h, which increased at 9.23% after 48 h and finally reached a cytotoxicity percentage of 10.61% after 72 h post-exposure.

### 3.2. Vascular Safety on the Chorioallantoic Membrane

**Figure 6:**
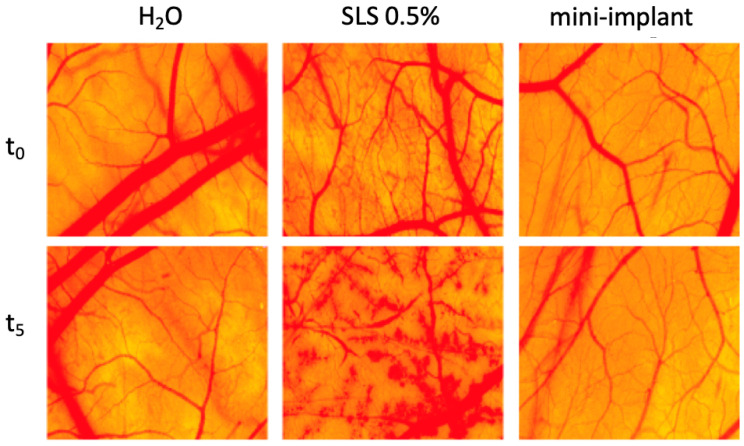
Irritation potential of mini-implant alloy extraction medium on the hen’s egg test on chorioallantoic membrane (HET-CAM) assay—before inoculation of sample/controls (t_0_) and after five minutes (t_5_) of contact with test sample/controls. H_2_O was used as negative control and sodium laurylsulphate (SLS) 0.5% was employed as positive control.

Compared to the positive control, which induced severe alteration of blood vessel architecture and functionality, with a high irritation score, the tested mini-implant alloy had a similar influence as the negative control, with no irritation (IS = 0) at the vascular level of the developing CAM (Figure 6). Furthermore, 24 and 48 h after the sample application, there was no sign of toxicity and the embryos showed a high viability rate. The capillary bed was developing similar to normal conditions with no interventions and the angiogenic process was active.

The extraction medium of the mini-implant alloy did not induce any alteration to the three parameters evaluated during the procedure, hemorrhage, coagulation or lysis, thus indicating optimal tolerability and compatibility on a vascularized mucosal-like tissue according to the scale proposed by Luepke (0–0.9—non-irritant, 1–4.9 weak irritant, 5–8.9/9.9 moderate irritant, 8.9/9.9–21 strong irritant) [42], as presented in Table 3.

## 4. Discussion

In this study, finite element models of the maxilla and the mini-implant were generated; orthodontic loading for en-masse retraction, intrusion and the pull-out test was simulated and the stress patterns generated by the implants were evaluated under different insertion angles and different CBT.

A self-drilling mini-implant prototype was chosen in the current study because it provides more bone contact, has higher initial stability than predrilling mini-implants and, in general, this type of mini-implant is recommended for the maxilla [46,47]. Sufficient primary stability is attainable by (i) altering the design of the mini-implant, (ii) increasing the length and diameter of the mini-implant [48,49,50], (iii) modifying the thread pitch [46] and (iv) changing the body shape (cylindrical, taper or combination designs) [28,51].

Using a custom-made mini-implant, this study investigated several factors that influence mini-implant stability and the correlation between them: CBT (1, 1.5 and 2 mm), insertion angle (30°, 60°, 90° and 120°) and force direction (axial, vertical and horizontal). Other studies investigated the correlation between CBT (1 and 2 mm) and insertion angle (30°, 45°, 60°, and 90°) [10]; between force type (traction and torsion), mini-implant design and insertion angle (45° and 90°) [52]; between insertion angle (60°, 90°, 120°), orthodontic force direction and exposure length of the mini-implant [13]; and between insertion angle (30°, 60° and 90°), force direction (60°, 90° and 120°) and CBT (1 and 2 mm) [53].

From a mechanical point of view, several studies suggest the multiple advantages of the perpendicular insertion of the mini-implant to the bone surface [10,11,54]. In this regard, our results showed that the stress decreases in the mini-implant by increasing the insertion angulation from 30°, 60° to 90° and increases again at 120°. Similar results were also reported by Zhang et al. [55]. Accordingly, Lin et al. found that an increase in implant insertion angle from 30° to 120° decreases stress on the bone [13] and Noble and collaborators revealed that the torque required for the removal of mini-implants inserted with an oblique inclination is greater than of those inserted perpendicular to the bone; this indicates that the stress levels decrease as the insertion angles increase (30° to 90°) [56]. However, given the availability of more appropriate bone for implant placement near the apical region of adjacent teeth, oblique insertion of orthodontic mini-implants was proposed to avoid root damage [57]. Insertion angle along with CBT has a significant impact on the primary stability of orthodontic mini-implants [58] and it has been stated that inserting the mini-implant at a 45°–60° angle to the bone surface provides greater stability against shear forces as compared with perpendicular insertion [26,59]. Wilmes also concluded that in order to achieve the best primary stability, an insertion angle of 60° to 70° is advisable [27]. Meanwhile, Moon et al. reported that mini-implants should be inserted with an angle of 70° to 80° to the long axis of the teeth for better stability and success in the posterior buccal region [60]. On the other hand, Caccifesta et al. [61] stated that, in order to enhance primary stability, the insertion angle should be kept stable during insertion and the threaded part should be inserted totally into the bone, obtaining an increased contact area between the mini-implant and the bone once the insertion angle decreases, but, at the same time, the cantilever load arm lengthens, resulting in 2 and 4 mm lever arms for the 90° and 30°, respectively. In addition, Butcher et al. revealed that applying the load 3 mm from the bone surface will enhance the screw failure rate better than if it has been applied 1 mm away from the bone surface [62].

Several studies investigated the relevance of the angle of insertion and the direction of force for the stability of mini-implants [12,13,63,64]. Concerning these aspects, the present paper studied three force directions: parallel to the longitudinal axis of the mini-implant (pull-out test), vertical and horizontal. Clinically, the direction of force is mostly perpendicular to the mini-implant (vertical and horizontal). However, the present results show that changing the direction of the force from horizontal to vertical leads to a decrease in the von Mises stress level of the implant and the direction of force has no significant effect on the cortical bone stress (*p* = 0.024). In good agreement with the above-mentioned results were also the ones reported by Lin et al. [13]. Nevertheless, another study [12] suggested an oblique loading direction instead of a perpendicular one. Still, this topic is a debatable one and there is no consensus in the literature; thus, the mechanical behavior of orthodontic mini-implants inserted at various angulations needs further investigation to clarify all the aspects involved in the orthodontic mini-implant success rate.

However, there are some limitations in the simulation of the present study: the properties of the material were assumed to be homogeneous; thus, no soft tissue was simulated and the interface between the bone and the mini-implant was considered to be as a frictionless contact. Nevertheless, these limitations do not influence the underlying mechanical mechanism.

Regarding the clinical biosafety of our newly developed orthodontic mini-implant, basic cytotoxic assays were performed by employing an in vitro model based on primary human gingival fibroblasts (HGF cells). These cells were chosen due to their direct exposure to any possible ion release from the orthodontic mini-implant [65], but also due to the fact that the gingival fibroblasts are the principal cells involved in the generation of the soft tissue that surrounds the orthodontic mini-implant [66]. Moreover, in vitro experiments are often preferred over in vivo ones due to minimal costs, time efficiency, reduced use of experimental animals but also due to the possibility of implementing well-controllable parameters [67]. However, all the in vitro experiments must be performed in agreement with ISO standard 10993-5:2009, like the ones employed in the current study.

The in vitro results obtained in the current study revealed that the metal alloy used for the development of the custom-made orthodontic mini-implant does not induce alterations of HGF cell morphology (Figure 5A) significative cell viability reduction (Figure 5B(i)) or potent cytotoxicity (Figure 5B(ii)). Thereby, according to ISO standard 10993-5:2009 [41] related to biological evaluation of medical devices, if the viability of the stimulated cells is not reduced by more than 30%, the test sample is considered non-cytotoxic. Thus, analyzing the results obtained in both colorimetric in vitro assessments (with the lowest cell viability rate of 87% and the highest cytotoxic rate of 10.61% after 72 h of treatment), the custom-made mini-implant alloy could be considered a biocompatible medical device and could be used safely for further in vivo investigations.

HET-CAM is an inexpensive, short-term in vivo method for predicting the ocular irritant effect of chemicals as an alternative to the classic Draize test in rabbits [18,68]. The irritation potential assessment can be a valuable method for the prediction of safety concerns in the development of bioengineered materials designed to be in contact with highly vascularized mucosa [69]. The designed mini-implant alloy, exposed to the perfused chorioallantoic membrane, showed no sign of irritation and no alteration of vascular parameters in the HET-CAM assay (Figure 6). Besides, the biocompatibility evaluation was performed for a significant number of compounds, including collagen and various types of dental polymeric implant materials; some studies analyzed bone regeneration [70,71]. In addition, vasculogenesis, a crucial process in implantology, can be assessed by the exposure to the chorioallantoic membrane [19] and our model showed that vessel development was not impaired by exposure to the mini-implant alloy extraction medium.

## 5. Conclusions

Present results revealed that oblique insertion provides adequate cortical bone engagement when the CBT is reduced. Stress distribution in the bone was high in the cortical, while very little stress was transmitted to the cancellous bone. In addition, it was significantly concentrated at the apex of the threads that were in contact with cortical bone.

When cortical bone thickness is reduced (1 mm), 30° of insertion is recommended in order to increase the contact area between the implant and the cortical bone. However, caution should be taken, as both the lever arm and the amount of threads exposed are increased as well. In this regard, future studies should assess the correlation between implant length, number of threads exposed and the insertion angle.

When cortical bone thickness is adequate (2 mm), an insertion angle of 90° is recommended as the 30° insertion angle generates a great deal of stress that can cause micro-fractures and necrosis in the cortical bone, leading to mini-implant failure.

Regarding the biosafety properties of the mini-implant, current in vitro and in vivo evaluations have shown that the sample extraction medium did not induce impairment of the HGF cell viable population or irritative potential when using the HET-CAM protocol, supporting thus the use of the custom-made mini-implant alloy in further experimental clinical applications.

## Figures and Tables

**Table 1:** ANOVA results of the pull-out force on bone’s body.

Source	SS	Df	MSS	F	*p*-Value
Angle	1763.792	3	587.931	517.457	*
CBT	436.389	2	218.194	192.04	*
Error	6.817	6	1.136		
Total	49,425.37	12			

CBT—cortical bone thickness; SS—Sum of squares; MSS—mean sum of squares; * *p*-value < 0.05.

**Table 2:** ANOVA results of the von Mises stress level on the implant and bone.

Source	SS	Df	MSS	F	*p*-Value
**BODY**					
Force Type	24,597.64	1	24,597.64	1131.11	0.000 *
Angle	43,644.5	3	14,548.16	668.99	0.000 *
Thickness	1627.86	2	813.93	37.428	0.000 *
Force Type*Angle	15,242.32	3	5080.77	233.64	0.000 *
Angle*Thickness	5173.8	6	862.3	39.65	0.000 *
Force Type*Thickness	32.576	2	16.28	0.75	0.512
Error	130.479	6	21.75		
Total	263,725.8	24			
**HEAD**					
Force Type	60,339.78	1	60,339.78	291.04	0.000 *
Angle	189,062.1	3	63,020.68	303.97	0.000 *
Thickness	3	2	6686.61	32.25	0.001 *
Force Type*Angle	72,134.79	3	24,044.93	115.97	0.000 *
Angle*Thickness	26,978.64	6	4496.44	21.69	0.001 *
Force Type*Thickness	92.41	2	46.21	0.223	0.807
Error	1243.96	6	207.33		
Total	812,244.9	24			
**CORTICAL**					
Force Type	920.49	1	920.49	8.915	0.024 *
Angle	118.25	3	39.42	0.382	0.77
Thickness	150.38	2	75.19	0.728	0.521
Force Type*Angle	1443.27	3	481.09	4.659	0.052 **
Angle*Thickness	747.89	6	124.65	1.207	0.413
Force Type*Thickness	140.81	2	70.41	0.682	0.541
Error	619.535	6	103.256		
Total	17,692.23	24			
**CANCELLOUS**					
Force Type	0.003	1	0.003	1.648	0.247
Angle	0.863	3	0.288	147.582	0.000 *
Thickness	1.44	2	0.72	369.38	0.000 *
Force Type*Angle	0.032	3	0.011	5.411	0.038 *
Angle*Thickness	0.594	6	0.099	50.817	0.000 *
Force Type*Thickness	0.008	2	0.004	2.029	0.212
Error	0.012	6	0.002		
Total	7.486	24			

SS—Sum of squares; MSS—mean sum of squares; * *p*-value < 0.05; ** *p*-value < 0.01.

**Table 3:** Classification of the mini-implant alloy irritation potential.

Sample	Irritation Score (IS)	Irritation Category
H_2_O	0	No irritation
SLS 0.5%	18.3	Strong irritation
Mini-implant	0	No irritation
